# From 3D Surface Documentation to Visualization in Forensic Practice: A Review of Current Methods and Emerging Technologies

**DOI:** 10.1111/apm.70215

**Published:** 2026-04-24

**Authors:** Haruki Fukuda, Lars Ebert, Florian Kreitmeier, Sebastian Eggert, Eva Meixner, Tadashi Hosoya, Wolf Schweitzer, Constantin Lux

**Affiliations:** ^1^ Institute of Forensic Medicine, Universität Zürich Zürich Switzerland; ^2^ Biometrics/Forensic Imaging, Zurich Forensic Science Institute Zurich Switzerland; ^3^ Department of Legal Medicine Gunma University, Graduate School of Medicine Maebashi Japan

**Keywords:** 3D documentation, 3D gaussian splatting, forensic autopsy, forensic investigation, photogrammetry

## Abstract

The accurate documentation of bodies, injuries, death scenes, accidents, and crime scenes is fundamental to forensic investigations. Traditional two‐dimensional (2D) photography and written reports have inherent limitations in representing complex spatial relationships. Recently, three‐dimensional (3D) surface documentation technologies, including laser‐scanning, structured light scanning, and photogrammetry, have been integrated into forensic practice to provide accurate, measurable, and reproducible digital evidence. This review provides an overview of current 3D methods and discusses their principles, advantages, and typical applications in documenting crime scenes and human remains. Furthermore, it introduces emerging image‐based reconstruction approaches, such as neural radiance fields (NeRF) and 3D Gaussian splatting (3DGS), which generate high‐quality reconstructions from multiple images. The integration of 3D data into immersive environments, such as virtual reality (VR) and augmented reality (AR), is also addressed, highlighting its potential in forensic analysis, courtroom presentation, and education. With the rapid evolution of 3D technologies, it is essential to understand the strengths and limitations of each technique in forensic applications. Future developments in algorithms and immersive integration will expand digital forensic capabilities.

## 1 Introduction

In the forensic field, accurate documentation of scene conditions, trace evidence, and the characteristics of injuries observed on the deceased and survivors are of paramount importance for further processing of evidence. Such documentation is essential not only to share visual evidence, reconstruct the circumstances of the incident, and provide evidence in court proceedings but also to enable subsequent re‐evaluation by other experts. Typically, forensic documentation is created through written reports supported by a series of two‐dimensional (2D) photographs, whose coverage is limited to what first appears relevant. In particular, photographic records allow for the visual assessment of specific features, such as tool marks, trace materials, and the morphology of injuries. An adherence to image and text formats that are 2D and easy to distribute through photocopying has both technohistorical and procedural reasons, in that criminal investigations and courts require evidence to be disclosable and reviewable by all parties. The sharing of such asynchronous channels is relevant; decoupling time constraints, such as any imposed immediacy, from a comprehensive review by all parties may be relevant, particularly for reasons of fairness [1].

However, 2D photography has limitations [2]. One focuses only on what appears relevant during photography when visiting a scene or examining a body. Even with comprehensive descriptive reports, recreating the overall spatial aspect of a crime scene or the anatomical distribution of multiple injuries may be impossible if all views, angles, and parts of the scene or body are not photographed. Despite the inclusion of scale bars, lens distortion and perspective errors can compromise the accuracy of retrospective measurements if the photographs are not taken orthogonally. Most critically, any such oversight during the initial documentation process is irreversible; missed evidence or latent injuries cannot be re‐examined once the scene or body has been altered. A comprehensive manner in which to obtain a full set of photographs is to target the creation of 3D models. This enforces a degree of completeness already at the level of 2D photos. The use of three‐dimensional (3D) reconstructed models allows an intuitive understanding of the spatial relationships of an entire body or crime scene. However, even before obtaining 3D models, the set of 2D photos required to create a good‐quality 3D model will cover far more angles, views, or parts of a scene or body than the typically limited 2D photo sets captured without the goal of 3D modeling. When a dozen or more photographs are typically taken, high‐quality 3D reconstruction generally requires hundreds of overlapping images [3].

Even if certain measurements are not obtained during the initial examination, arbitrary distances and dimensions can be measured using a 3D model. Furthermore, 3D representation may provide additional depth information that cannot always be obtained from 2D images alone, enabling a more detailed re‐evaluation of injury morphology [4]. Additionally, the registration and alignment of various 3D datasets enables investigators to correlate evidence from different sources, providing a comprehensive spatial context for even complex forensic reconstructions.

The utility of 3D models in forensic investigations has been demonstrated [5, 6, 7, 8, 9, 10, 11, 12, 13, 14]. 3D data can be used for computer‐based evaluations [15, 16], simulations, autopsy training [17], education, and reconstruction within virtual reality (VR) environments [18, 19, 20], as visual aids for presenting evidence in court [21], and VR‐based applications, including collaborative expert case reviews, the acquisition of witness testimony, and the presentation of evidence in court [8]. Several techniques are available for 3D surface documentation, including photogrammetry, laser scanning, and structured light scanning (SLS) [22, 23]. In addition, postmortem imaging modalities such as postmortem computed tomography (PMCT) and magnetic resonance imaging (PMMR) primarily provide internal anatomical information, but can also visualize the surface [24]. In recent years, novel approaches based on radiance fields, such as 3D Gaussian Splatting (3DGS) and neural radiance fields (NeRF), have emerged [25, 26, 27, 28, 29]. Understanding the characteristics and differences among these technologies is important for selecting appropriate methods in forensic applications.

This review provides an overview of major techniques used for 3D surface documentation and forensic rendering. The characteristics, advantages, and limitations are summarized and recently‐developed approaches for 3DGS and NeRF are introduced. Although the primary focus of this review is 3D surface documentation techniques, related technologies, such as postmortem imaging and immersive visualization, are also briefly discussed where they complement surface based forensic documentation and interpretation.

## 2 Techniques for 3D Surface Documentation

In forensic science, the commonly used techniques for 3D surface documentation include photogrammetry, laser scanning, and SLS. More recently, novel approaches, such as 3DGS and NeRF, have also been applied. Laser scanning, which projects a laser beam onto an object, and SLS, which projects patterned light onto a surface, are classified as active scanning techniques [30, 31, 32]. In contrast, photogrammetry is considered a passive scanning method in which a large number of photographs are captured from different angles around an object and processed using photogrammetry software to establish the correspondence between images and reconstruct the 3D structure of the object. Approaches such as 3DGS and NeRF can also be regarded as image‐based methods that rely on multiple photographs of the target object. In addition, postmortem imaging modalities such as PMCT and PMMR, which enable the evaluation of internal findings, are briefly mentioned as complementary approaches to surface documentation.

### 2.1 Laser Scanning

Laser surface scanners operate by projecting a laser beam onto an object and gradually scanning across the surface. The reflected light is captured by optical sensors, permitting the geometry of an object to be reconstructed [33]. Using laser scanners, complex scenes can be rapidly reconstructed with high geometric accuracy by generating dense point clouds [22]. Therefore, laser scanning is a valuable tool for documenting and preserving crime scenes. In forensic practice, it has been applied to the investigation of crime scenes, human remains, including skeletonized bodies, and various types of forensic evidence, such as weapons and personal items (e.g., accessories, dental prostheses, or dental models) [18, 34, 35]. However, laser scanning is sensitive to motion artifacts. The scanned object must remain stationary for a relatively long period because even a slight movement during data acquisition may result in distortions of the collected point clouds [36].

Another limitation is that the resulting 3D model may not contain color information of the original surface texture unless the device is equipped with an integrated optical camera system (e.g., NextEngine) or texture information is subsequently applied using appropriate software such as MeshLab [33]. In addition, potential safety concerns have been raised regarding the exposure of sensitive tissues such as the retina to laser light [32]. Consequently, the use of this type of scanner on living human subjects is uncommon, although specialized laser systems designed for human‐body scanning do exist [36]. Furthermore, digitizing transparent and dark surfaces is difficult as laser light tends to be absorbed by these materials [37, 38].

### 2.2 LiDAR Scanning on Mobile Devices

Laser‐scanning systems are generally expensive and require specialized equipment. However, since autumn 2020, several consumer devices, such as certain models of iPhones and iPads (Apple Inc., Cupertino, CA, USA), have been equipped with integrated light detection and ranging (LiDAR) sensors. These devices are being increasingly investigated as affordable, consumer‐level scanners for forensic applications [39, 40].

LiDAR sensors determine the distance between the sensor and surface of an object by emitting infrared light pulses and measuring the time required for the reflected light to return to the sensor. Based on the time‐of‐flight principle, the system can estimate the spatial positions of surfaces and generate 3D representations of the surrounding environment [39].

One of the primary advantages of LiDAR‐based scanning is its high speed. For example, Desai et al. demonstrated that an iPad equipped with a LiDAR sensor was able to complete a full scan of a scene within approximately 15 min, whereas scanning the same scene using a professional‐grade terrestrial laser scanner required several hours to achieve the level of accuracy required for legal documentation [40, 41]. In terms of accuracy, Desai et al. reported that the root mean square error (RMSE) of the profile distances obtained between handheld (iPad‐based) scans of a damaged vehicle and survey‐grade terrestrial laser scanning (TLS) was approximately 3 cm, which was considered acceptable for forensic purposes [41]. Similarly, Kottner et al. evaluated distance measurements in different environments and reported errors of less than 0.10 cm in a simulated crime scene, < 0.69 cm in a garage environment, and < 0.17 cm for a parked vehicle. The overall mean absolute error (MAE) and standard deviation (SD) were reported as 0.22 cm and 0.18 cm, respectively, indicating relatively small variations in measurement accuracy [39].

However, similar to conventional laser scanners, LiDAR‐based systems may encounter difficulties when scanning reflective or transparent surfaces, such as mirrors, glossy objects (e.g., evidence markers), or glass surfaces (e.g., vehicle windows). In addition, dark or light‐absorbing materials, such as black fabrics, gloves, and socks, may reduce scanning accuracy. These limitations are common to many optical imaging systems [37, 39]. Furthermore, the use of consumer communication devices, such as smartphones or tablets, for forensic documentation may raise concerns about data security and confidentiality, which can complicate their adoption in certain forensic environments.

### 2.3 Structured Light Scanning (SLS)

SLS operates by projecting patterned light onto the surface of an object, typically in the form of striped planes. The resulting distortions can be analyzed to reconstruct the 3D shape of the surface because the projected pattern is deformed according to the object geometry [31]. Unlike laser scanning, structured light systems can simultaneously capture nearly the entire field of view. This allows the surface geometry to be recorded in a relatively short acquisition time and makes the method less susceptible to motion artifacts [32, 42]. In addition, SLS does not raise significant safety concerns and poses minimal risks to the subjects. Therefore, structured light systems are frequently used to document injuries in living victims [32].

SLS can also be applied to deceased individuals. This technique has been used to generate 3D models of skeletal remains, and SLS can provide higher accuracy than laser scanning when applied to bone surfaces [24, 43]. SLS has been used to document surface injuries in living individuals and for recording objects, particularly for the analysis of patterned injuries that correspond to specific sources, such as shoeprints, bite marks, bruises, or impressions from objects [44]. SLS has also been utilized in forensic anthropological research [9, 45, 46]; for example, in age estimation based on pelvic morphology [9].

One limitation of SLS is that, unlike laser scanning, its performance is generally dependent on ambient light conditions [47]. In addition, the measurable scanning range is generally limited to medium sized objects, making the technique less suitable for large‐scale documentation such as crime scene reconstruction. Similar to laser scanners, SLS has limitations when scanning reflective (specular), semi‐transparent, transparent, or very dark surfaces [48, 49], although these issues may be mitigated by applying a spray coating to the object prior to scanning [48].

### 2.4 Photogrammetry

Photogrammetry is a scientific method that uses photographs to generate 3D models and obtain accurate measurements of terrain, infrastructure, or objects with defined volumes. This field originated in the 19th century, following the invention of photography and its subsequent application in cartography and topography [50]. With the development of modern computers and photogrammetric software, this technique has become increasingly accessible and is now widely used in various fields, ranging from cultural heritage preservation to medical applications [51, 52]. Compared with laser scanning or SLS, photogrammetry is relatively simple to implement because it requires only a digital camera and appropriate processing software.

Several commercial software packages are used, including PhotoModeler (Eos Systems Inc., Vancouver, Canada), 3DF Zephyr (3Dflow SRL, Verona, Italy), RealityScan (Capturing Reality, Bratislava, Slovakia), and Agisoft Metashape (Agisoft LLC, St. Petersburg, Russia). In addition, open‐source solutions such as Meshroom (AliceVision) are available.

The advantage of photogrammetry is the relatively short acquisition time required to capture the images, which may vary depending on the size of the documented object. For example, Ujvári et al. reported that scanning a human‐body using laser scanning from five scanning positions required approximately 20 min, whereas photogrammetry was considerably faster, with the slowest photogrammetric workflow requiring only 8–9 min. Their results also indicated that photogrammetric models were almost as accurate as models generated using certain laser scanning systems [2].

The quality of photogrammetric models depends on several factors, including the resolution of the original images, photogrammetry software used [53], sufficient overlap between images, and appropriate lighting conditions. A standardized photographic workflow combining synchronized high‐performance studio flash units with fixed camera settings has been used to acquire large image datasets, typically consisting of several hundred photographs within a few minutes. This approach provides uniform illumination, short exposure times, and reproducible image quality and has been shown to be highly suitable for detailed surface reconstruction in forensic practice [3].

However, photogrammetry has some limitations. Surfaces lacking distinctive features, deep surface depressions, body hair, highly‐reflective moist surfaces, or surfaces covered with body fluids may generate artifacts and complicate accurate reconstruction [2, 33, 54, 55]. Photogrammetry relies on triangulation to reconstruct 3D geometry from multiple images. Therefore, reconstruction becomes challenging when reliable feature points cannot be detected or when the surface areas exceed the effective triangulation angles between images, leading to difficulties in image registration [56].

Another limitation of photogrammetry is that image acquisition is typically conducted manually. Consequently, capturing photographs at consistent angles across different cases may be difficult. Additionally, if it becomes apparent that the number or coverage of images is insufficient after reconstruction, it is often difficult to repeat the image acquisition process. To address this issue, multicamera systems have been developed in which multiple cameras capture images simultaneously from different angles. Because the images are acquired almost instantaneously, such systems can obtain the photographs required for 3D reconstruction even in living subjects who may have difficulty remaining completely still for extended periods [57, 58].

In crime scene reconstruction, photogrammetry using unmanned aerial vehicles (UAVs) is sometimes used, for example, in large‐scale scenes, such as traffic accidents or forest fires [59, 60, 61]. UAV technology has become increasingly affordable in recent years and can be particularly useful in situations where direct human access to a scene is difficult.

### 2.5 Radiance Field‐Based Methods (NeRF and 3DGS)

**FIGURE 1:**
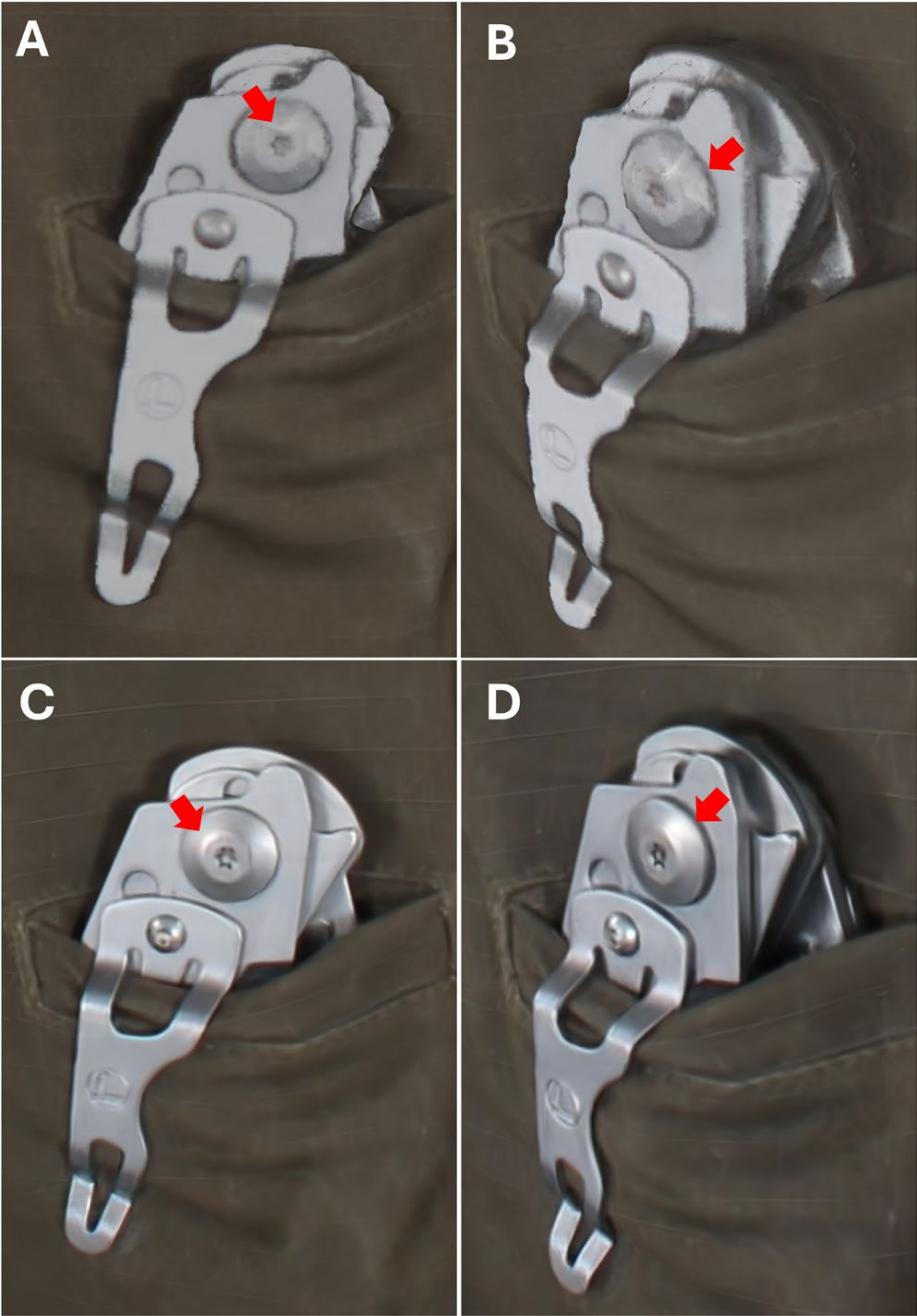
Comparison of metal fitting reconstruction between (A, B) photogrammetry and (C, D) 3D Gaussian splatting (3DGS). Both models were generated from the same dataset. (A, C) Front view; (B, D) oblique right view. The 3DGS reconstruction, which appears sharper, more accurately reproduces view‐dependent light reflections when the viewing perspective is altered (red arrows) than photogrammetry.

Radiance field‐based methods have recently emerged as approaches for synthesizing new viewpoints of scenes captured from multiple photographs or videos. NeRF, introduced by Mildenhall et al., utilizes a deep neural‐network to represent a scene as a continuous 3D function. By processing a set of 2D images, the network learns to predict the volume density (σ) and view‐dependent color (RGB) for any specific coordinate in space. The resulting volume dataset is then visualized using volumetric ray‐casting algorithms, enabling better synthesis of novel viewpoints that accurately capture complex light transport, such as specular reflections and highlights, on moist or metallic surfaces compared to photogrammetric model [1, 25]. A comparison between photogrammetry and 3DGS is shown in Figure 1. Photogrammetric reconstructions (Figure 1A,B) appear less sharp and show limited reproduction of view‐dependent reflections, whereas 3DGS reconstructions (Figure 1C,D) provide sharper details and more realistic light reflections when the viewing perspective is altered.

More recently, 3DGS has been proposed as a volume rendering technique that directly represents 3D scenes using a set of Gaussian distributions [26, 28, 62]. NeRF is limited by relatively slow training times. In contrast, 3DGS enables substantially faster training and supports real time rendering.

In forensics, research on the application of radiance field‐based methods has emerged [27, 28, 63]. The advantages and limitations of NeRF and photogrammetry using human remains and mock crime scenes have been compared. NeRF produced better reconstruction results than photogrammetry for objects with monochromatic surfaces and limited textures, as well as for materials that are metallic, reflective, or semi‐transparent [27]. Rangelov et al. conducted a comparative study of NeRF and 3DGS in indoor and outdoor environments. Although NeRF is a powerful tool for generating high‐quality reconstructed images, it was associated with high computational demands and was particularly sensitive to challenging lighting conditions and occlusions, especially in indoor scenes. In contrast, 3DGS provides a more efficient and robust alternative, substantially reducing processing time while producing reconstructed images with comparable or superior visual quality and fewer visual artifacts [64].

Applications of 3DGS to crime scene reconstruction have also been reported. Chor et al. demonstrated that large objects, such as desks and doors, showed stable reconstruction accuracy, whereas smaller or thinner objects such as bloodstains exhibited increased relative errors due to scale‐related artifacts. Nevertheless, the absolute physical accuracy was consistent. Their quantitative analysis showed that the measurement errors in the main experiment were 1.73–3.58 mm on average, which falls well within the acceptable range of conventional manual measurements [28]. However, this study had several limitations. Currently, converting radiance field representations into high‐quality mesh models is challenging. Because this technology is relatively new, issues such as compatibility with other software packages, optimal image acquisition conditions, and standardized capture protocols have not yet been sufficiently investigated.

Overall, these results highlight the potential of 3DGS as a reliable and practical tool for digital preservation and reconstruction of crime scenes. This suggests that radiance field‐based methods have considerable potential for future application in the scanning of human remains and forensic objects.

### 2.6 Postmortem Computed Tomography (PMCT) and Magnetic Resonance Imaging (PMMR)

PMCT is widely used in forensic investigations because it enables non‐destructive examination of internal anatomical structures [65, 66, 67, 68, 69]. In addition, volume‐rendering techniques allow certain aspects of the body surface to be visualized, for example, for the evaluation of external injuries such as stab wounds using global illumination rendering [70] or for the generation of 3D models of bones, skin, internal organs, and sometimes objects [20, 71, 72, 73]. Micro‐CT can also be used to obtain highly detailed models of small specimens [74]. PMMR is an additional imaging technique that was introduced into forensic practice in the early 2000s [7, 75, 76, 77]. As with PMCT, PMMR datasets can be used to reconstruct 3D models, enabling quantitative analyses, such as measurements of organ volumes (e.g., the hippocampus) [78] or bone thickness [79]. Although visualization of surface structures is theoretically possible, its application in surface documentation has received little attention. To facilitate a multimodal analysis, PMCT and PMMR data can be transformed into polygon models and fused with surface documentation. For such volumetric datasets, maintaining a minimum resolution of 1.5 mm (or ideally submillimeter) in all three dimensions is essential to ensure the generation of high‐quality, anatomically‐accurate 3D models and to avoid interpolation artifacts during reconstruction [80]. PMCT‐ and PMMR‐based reconstructions do not capture the color information of the surface, and metal artifacts may occur when metallic objects are present. In addition, CT and MRI systems are expensive and require specialized equipment.

## 3 Data Representation

### 3.1 Point Clouds

**FIGURE 2:**
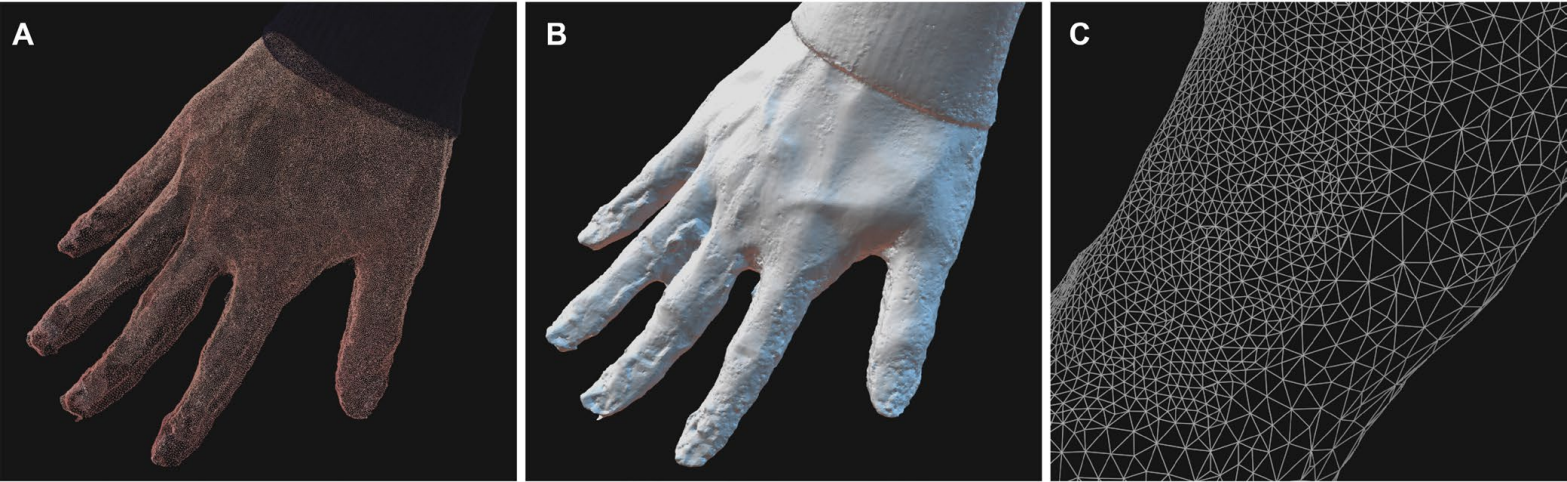
Point cloud and mesh representations of a reconstructed hand model. (A) Point cloud, (B) polygonal mesh, and (C) magnified view of the fingers in the polygonal mesh displayed in wireframe mode, illustrating the triangular structure of the mesh.

Laser and structured light scanners typically acquire the surface geometry directly in the form of point clouds, which consist of a large number of spatial coordinates representing the sampled surface points. In photogrammetry, point clouds are not measured directly but are reconstructed computationally using structure‐from‐motion (SfM) algorithms that estimate both camera positions and 3D point locations from overlapping images [81]. NeRF and 3DGS typically use camera poses and sparse point clouds estimated through structure‐from‐motion pipelines (e.g., COLMAP) to initialize scene representations. An overview of point cloud and mesh representations is shown in Figure 2. A point cloud representation is illustrated in Figure 2A.

### 3.2 Polygon Meshes

Polygon meshes consisted of 3D points called vertices, which are interconnected by edges to form polygons. Examples of polygon mesh representations are shown in Figure 2B,C. In many 3D reconstruction workflows, meshes are generated from point cloud data using surface reconstruction algorithms. In point cloud data, noise and artifacts may be introduced during the scanning or reconstruction process, and unwanted points must often be removed manually or through automated filtering. In photogrammetry workflows, texture information derived from photographs can be projected onto a reconstructed mesh surface to generate textured mesh models. Converting point cloud data into polygonal meshes enables various applications, including 3D printing, structural analysis, and visualization in virtual reality environments [31, 66].

### 3.3 Volumetric Data (CT and MRI)

CT and MRI volumes consist of individual elements called volume pixels (voxels), which are analogous to pixels in two‐dimensional images. Each voxel has a spatial position within the volume and contains a value depending on the imaging modality [66, 82]. In most medical imaging viewers, CT and MRI datasets are visualized using volume rendering techniques (VRT), in which voxel values are mapped to color and opacity to enable 3D visualizations [83]. Recent rendering approaches simulate the physical behavior of light, enabling realistic optical effects, such as depth‐of‐field and shadows. Although these techniques can improve visual realism and depth perception, they typically have higher computational costs and slower rendering performance [66]. Volumetric datasets can also be converted into polygonal mesh models, which enable applications such as 3D printing, detailed analysis, and presentation in court [66, 84].

### 3.4 Radiance Scene Representations

NeRF represents a scene as a continuous volumetric function that predicts the color and density of any point in space [25]. Neural‐network‐based formulations require substantial computational resources, which can make model manipulation and direct editing of scene elements challenging. In contrast, 3DGS represents scenes using a collection of anisotropic Gaussian primitives distributed throughout the 3D space [26]. This approach builds upon earlier research on splatting‐based rendering techniques, including the splatting algorithm proposed by Westover and subsequent developments such as EWA splatting, which established the basis for modern point‐based rendering methods [85, 86, 87]. One of the major advantages of 3DGS is its ability to efficiently render complex visual effects, such as view‐dependent lighting and transparency. Because a scene is represented explicitly by Gaussian primitives rather than an implicit neural field, the representation can be more easily edited and integrated with other common 3D formats.

Many 3DGS implementations store Gaussian parameters in widely‐supported formats, such as PLY files, facilitating inter‐operability with existing visualization and processing tools [88]. By importing the data into dedicated editors, such as the open‐source, SuperSplat Editor (PlayCanvas Ltd., London, UK), additional operations such as distance measurements can also be conducted. Although early implementations produced relatively large datasets, recent studies have proposed compression techniques to reduce storage requirements [87].

## 4 Discussion

### 4.1 Comparison of 3D Surface Documentation Techniques

3D reconstruction technologies have evolved steadily over time, ranging from traditional photogrammetry to more recent approaches, such as 3DGS. Each technique has advantages and limitations. Therefore, it is essential to understand the characteristics of each method and select the most appropriate technique for specific forensic applications. As summarized in Table 1, each technique has distinct strengths and limitations depending on the forensic application. Laser scanning and SLS provide high geometric accuracy but are limited by surface properties and acquisition constraints. Laser scanning typically relies on time‐of‐flight or phase‐shift measurements and is well suited for large‐scale environments, whereas SLS projects patterned light and is more suitable for high‐resolution documentation of smaller objects or injuries. Photogrammetry enables flexible and cost‐effective data acquisition with high‐quality texture but depends on sufficient image overlap and surface features. NeRF and 3DGS enable highly realistic rendering and improved handling of challenging surfaces but still face challenges in terms of standardization and integration into forensic workflows. In contrast, PMCT and PMMR provide valuable internal anatomical information but are limited in photorealistic surface representation.

**TABLE 1:** Comparison of major techniques for 3D surface documentation.

Technique	Principle	Advantages	Limitations
Laser scanning	Laser beam is projected onto the object surface and the reflected signal (either by time of flight or phase‐shift) is used to generate a dense point cloud representing the geometry of the scene	High geometric accuracy; suitable for documenting large environments; fast	Sensitive to motion artifacts; expensive equipment; color texture may require additional processing; difficulties with dark or reflective/refractive surfaces
LiDAR (mobile devices)	Time‐of‐flight measurement of emitted infrared pulses to estimate distances between the sensor and surrounding surfaces	Rapid acquisition; portable; relatively low‐cost; increasingly available in consumer devices	Significantly lower accuracy than professional scanners; difficulties with reflective, transparent, or dark surfaces; potential data security concerns
Structured light scanning (SLS)	Projection of patterned light onto the object surface; deformation of the projected pattern is analyzed to reconstruct 3D geometry	High precision to medium sized objects; fast acquisition; suitable for documenting injuries in living subjects	Sensitive to ambient lighting conditions; limited scanning range; less suitable for large scenes, slow
Photogrammetry	Multiple overlapping photographs are processed using feature matching and triangulation to reconstruct a fully textured 3D model	Low‐cost; flexible acquisition; high‐quality texture information; suitable for bodies, objects, and scenes; the ground truth is a complete set of 2D photographs that as such can be invaluable	Requires sufficient image overlap and surface texture; reflective or featureless surfaces may cause artifacts; acquisition is often manual
Radiance field‐based methods (NeRF/3DGS)	Image‐based neural rendering approaches that learn volumetric scene representations from multiple photographs to synthesize novel viewpoints	High visual realism; capable of reconstructing reflective or texture‐poor surfaces; efficient rendering (especially with 3DGS); the ground truth is a complete set of 2D photographs that as such can be invaluable	Computational requirements; limited standardization; conversion to mesh models remains challenging; while it provides measurement tools, the methodology is not yet matured
PMCT/PMMR	Tomographic imaging techniques that reconstruct internal anatomy using X‐ray CT or magnetic resonance imaging	Non‐destructive examination of internal structures; volumetric datasets; data conversion to surface polygons possible	No photorealistic surface texture representation; expensive equipment; requires specialized facilities; low surface resolution

Combining multiple techniques is an important aspect of 3D documentation. For example, because TLS itself does not produce color, this limitation can be compensated for by integrating images captured with a built‐in camera or by combining TLS with additional photogrammetric image acquisition. Such multimodal approaches enable efficient collection of high‐quality 3D data [89, 90]. All optical documentation methods, whether active or passive, face considerable challenges when capturing dark, reflective, or refractive surfaces. Although these limitations can be mitigated through the application of matting sprays to ensure geometric accuracy, this comes at the expense of surface color information because the spray obscures the original texture. 3D datasets also facilitate the integration of multiple imaging modalities. Chiara et al. reported a rapid workflow for combining CT scanning data with external photogrammetry [5]. By integrating internal CT information with external surface data, the resulting visualization can provide a clearer and more intuitive representation of the findings. Such multimodal reconstructions have potential applications in courtroom presentations [8].

### 4.2 Immersive Applications: Virtual and Augmented Reality (AR) in Forensic Practice

One of the most promising applications of 3D reconstruction technologies is their integration into immersive visualization environments. Unlike conventional 2D images, 3D models can be explored interactively using VR and AR systems. Immersive technologies such as VR and AR are increasingly used in a wide range of fields, including entertainment, gaming, healthcare, and medical education [91, 92, 93]. In VR environments, users interact with a completely virtual scene through head‐mounted displays, whereas AR overlays digital information onto a user's real‐world environment [94]. Head‐mounted displays (HMDs), represented by devices such as MetaQuest 3, as well as AR functions integrated into tablets and smartphones, such as iPads and iPhones, have become increasingly accessible. Because of their accessibility, their applications have expanded across various fields, including medicine [95, 96].

In forensics, VR has been used for the reconstruction and exploration of virtual crime scenes, such as the concept of a “forensic holodeck” [8]. VR‐based environments permit 3D crime scenes to be visualized and presented to investigators, authorities, and witnesses. VR can serve as a tool for forensic experts to conduct 3D reconstructions and analyses. VR has been used to document expert assessments or witness testimonies during virtual examinations of digitally‐reconstructed crime scenes. In Switzerland, VR‐based reconstruction has also been used in actual court proceedings. Rinaldi et al. showed that crime scene reconstructions generated through photogrammetry and visualized in VR can support the examination of fire scenes and enhance forensic decision‐making [97].

AR‐based visualization has also been investigated in forensic practice to providing additional imaging information during autopsies [11, 98]. For instance, a tablet‐based AR system was used to overlay surface information of the body with CT‐derived data in real time, enabling investigators to visualize the spatial relationship between external findings and internal anatomical structures [98]. Bulliard et al. demonstrated the use of an HMD, such as the Microsoft HoloLens, to visualize PMCT data directly within the user's field of view during autopsy [99]. Fukuda et al. demonstrated that CT‐based skeletal models visualized using tablet‐based AR could assist in estimating the origin and direction of injury [100].

### 4.3 Future Perspectives and Emerging Technologies

Radiance field‐based reconstruction techniques, such as NeRF and 3DGS, are still developing technologies, and several aspects require further investigation. Future studies should focus on evaluating the reconstruction accuracy across different algorithms, comparing these techniques with established methods such as photogrammetry, and improving inter‐operability with existing software tools. Recent advancements, such as R^2^‐Gaussian [101], demonstrate the potential of 3DGS in tomographic reconstruction by addressing integration biases in X‐ray data, enabling a more accurate and efficient recovery of internal structures directly from volumetric projections.

In addition, data acquisition using mobile devices may involve automatic synchronization, cloud‐based processing, or storage in geographically distributed servers, potentially complicating data protection compliance and chain‐of‐custody management.

Furthermore, the use of open‐source or community‐driven software tools, which are frequently adopted in emerging reconstruction workflows, may introduce additional security vulnerabilities and reproducibility concerns if not properly validated and controlled.

Future forensic workflows may increasingly integrate multiple data sources, including 3D reconstructions of bodies, objects, and crime scenes. These integrated datasets enable a more comprehensive digital reconstruction of forensic events. However, implementation of such integrated forensic datasets may present practical challenges. Combining crime scene documentation with autopsy findings often requires collaboration between multiple institutions, such as forensic pathology departments and law enforcement agencies. Access to and integration of such data may be restricted, depending on legal frameworks and data protection regulations.

## 5 Conclusion

3D surface‐documentation techniques are becoming increasingly important in forensic investigations because they provide accurate and reproducible representations of bodies, objects, and crime scenes. Although established methods such as laser scanning, SLS, and photogrammetry are still widely used, emerging approaches such as neural rendering techniques, including NeRF and 3DGS, offer new possibilities for high‐quality reconstruction and visualization. The integration of these 3D models into immersive environments, such as virtual and augmented reality, may enhance forensic analysis, education, and courtroom presentations. Continued technological development, combined with standardized acquisition workflows and improved software inter‐operability, will likely expand the role of 3D reconstruction and visualization in forensic practice in the coming years.

## Funding

This work was supported by the following grants: Overseas Research Fellowships (grant number 202560103) from the Japan Society for Promotion of Science (JSPS), the Gunma Prefecture Physician Training and Overseas Study Support Program (2025).

## Ethics Statement

The authors have nothing to report.

## Conflicts of Interest

The authors declare no conflicts of interest.

## Data Availability

The data that support the findings of this study are available from the corresponding author upon reasonable request.
